# Prenatal Exposure to Wood Fuel Smoke and Low Birth Weight

**DOI:** 10.1289/ehp.10782

**Published:** 2008-01-15

**Authors:** Amna R. Siddiqui, Ellen B. Gold, Xiaowei Yang, Kiyoung Lee, Kenneth H. Brown, Zulfiqar A. Bhutta

**Affiliations:** 1 Department of Public Health Sciences, University of California, Davis, California, USA; 2 Department of Community Health Sciences, Aga Khan University, Karachi, Pakistan; 3 College of Public Health, University of Kentucky, Lexington, Kentucky, USA; 4 Department of Nutrition and Program in International and Community Nutrition, University of California, Davis, California, USA; 5 Department of Pediatrics and Child Health, Aga Khan University, Karachi, Pakistan

**Keywords:** birth weight, cooking habits, historical cohort, natural gas, pregnancy, propensity scores

## Abstract

**Background:**

Maternal exposure to wood fuel smoke may lead to impaired fetal growth due to hypoxia and or oxidative stress from smoke constituents such as carbon monoxide and particulate matter.

**Objectives:**

We studied the risk of low birth weight (LBW) and reduced mean birth weight in relation to reported use of wood for cooking during the prenatal period, compared with natural gas (NG).

**Methods:**

We studied a historical cohort of women who had a singleton live birth in the years 2000–2002, from a semirural area of Pakistan. Infant’s birth weight was obtained from records, and prenatal records had data for maternal body mass index and parity. Cooking habits, daytime sleep habits, and type of fuel used during the pregnancies in 2000–2002 were ascertained by a survey done in 2004–2005. We performed multiple linear and logistic regression modeling using propensity scores to adjust for confounding variables.

**Results:**

Unadjusted mean (± SD) birth weight was 2.78 ± 0.45 kg in wood users, and 2.84 ± 0.43 kg (*p* < 0.06) in NG users. Infants born to wood users averaged 82 g lighter than infants born to NG users when weight was adjusted for confounders (*p* < 0.07). The rate of LBW (< 2,500 g) was 22.7% among wood users compared with 15.0% in NG users (*p* < 0.01), for an adjusted relative risk of 1.64 (95% confidence interval, 1.10–2.34). The population attributable risk for LBW explained by wood use was estimated to be 24%.

**Conclusion:**

Cooking with wood fuel during pregnancy, a potentially modifiable exposure, was associated with LBW and marginally lower mean birth weight compared with using NG.

Low birth weight (LBW) is associated with high mortality and morbidity in infants (Ismail et al. 2003; Khan et al. 2005; Moss et al. 2002; Tripathy et al. 2002). LBW leads to postnatal stunting (Fikree et al. 2000), micronutrient deficiencies (Rasmussen 2001), impaired psychomotor development (Tandon et al. 2000), elevated rates of morbidity (Moss et al. 2002), and chronic disease (Barker et al. 2002). The overall rate of LBW in Pakistan is 19% [UNICEF (United Nations Children’s Fund) 2006]; hospital-based urban data show rates ranging from 12 to 42% (Ismail et al. 2003; Khan et al. 2005) and field-based data from rural areas indicate rates of up to 31% [World Health Organization (WHO) 2007].

In developed countries, smoking is the leading cause of LBW, followed by low maternal weight gain during pregnancy (Kramer 1998). In Pakistan, the prevalence of smoking in women is reported to be low (3.5%) (Nasir and Rehan 2001); use of inhaled (*huqqa*) and chewing tobacco, however, is not uncommon in rural areas (Khan and Siddiqui 2002). Up to 70% of households use wood, biomass, and/or crop residues as cooking fuel, whereas 53% of households use wood alone as cooking fuel (Rehfuess et al. 2006; WHO 2005).

Fetal intrauterine growth retardation due to smoking (Centers for Disease Control and Prevention 2002) is partly explained by placental hypoxia induced by carbon monoxide, which depresses energy-dependent processes, leading to insufficiencies in amino acid transport (Sastry 1991). Transplacental transfer of polyaromatic hydrocarbons from ambient air pollution and environmental tobacco smoke from mother to fetus also has been reported (Perera et al. 1999). Similarly, wood smoke exposure during pregnancy can lead to impaired fetal tissue growth through hypoxia and/or oxidative stress resulting from its constituents, including CO and particulate matter (Kourembanus 2002; Li et al. 2003). Elevated risks for LBW associated with outdoor air pollution have been reported from other settings (Dejmek et al. 1999; Lee et al. 2003; Maisonet et al. 2001; Salam et al. 2005; Wang et al. 1997; Wilhelm and Ritz 2005). Two studies, one from Guatemala (Boy et al. 2000) and one from Zimbabwe (Mishra et al. 2004), have reported associations of use of wood fuel with LBW, and a 63- to 120-g reduction in mean infant birth weight. Because of the complex, multifactorial etiology of LBW and potential confounding from demographic, nutritional, reproductive, and socioeconomic factors, the evidence for the effect of ambient air pollutants is not considered adequate (Glinianaia et al. 2004; Maisonet et al. 2004). The importance of examining this hypothesis also stems from other facts; for example, the incidence of LBW is high in the region, and biomass use in the country is high. In addition, the proportion of births that are LBW could be grossly underreported because a large proportion of infants are not weighed at birth.

We therefore undertook a population-based study to examine risk of LBW and mean birth weight in relation to use of wood fuel during the prenatal period, while controlling for these potentially important confounding factors.

## Methods

The study protocol was approved by the institutional review boards at the University of California, Davis, and the Aga Khan University (AKU) in Karachi. The study area was Rehri Goth, a poor, semirural community located 50 km from Karachi. All of the defined area was included in the house-to-house survey when enrolling pregnant women, 2000–2002, and the same method was adopted in the survey in 2005. Traditional birth attendants were the main source of maternal care during pregnancy and labor.

### Study setting

Rehri Goth is a coastal fishing village in Pakistan with a population of > 35,000 residents. More than 40% of the population is poor, using the definition of having an annual income of 625 rupees (Rs) per capita [International Union for Conservation of Nature (IUCN) 2003]. The area is deficient in basic utility services, transportation, and health services (Hasan 2002; IUCN 2003). More than 60% of men and 90% of women have received no formal schooling or education (IUCN 2003). Educational institutions beyond the primary and secondary levels are not present in the area. Primary health care is provided through a rural health center, mainly for immunization and minor clinical problems. The main occupation of the residents is fishing. About half of the population uses wood as fuel for cooking and heating (Hasan 2002).

### Selection of study sample

All married women 15–45 years of age who delivered a singleton live birth during 2000–2002 were included. Pregnancies were identified by interviewing women during house-to-house surveys in 2000–2002.

**Figure 1:**
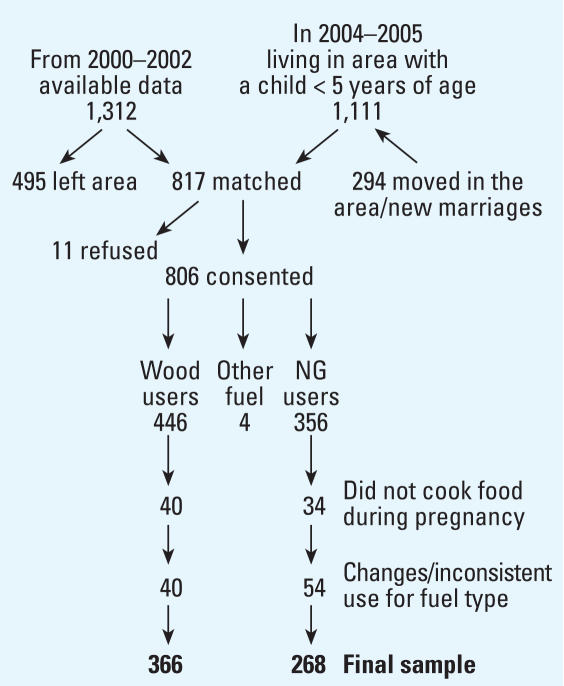
Attainment of final sample size.

A total of 1,111 women with at least one child < 5 years of age were identified in the area. A total of 817 of these women could be matched with birth record data at AKU (Figure 1) by using specific identification numbers; names of the head of the household, participant, and spouse; age of the child; house address; and neighborhood. Any pregnancy that ended during 2000–2002 was defined as the index pregnancy; this was the last pregnancy when a woman delivered twice during this period.

### Interview in 2004–2005

The questionnaire was pilot tested in a sample of women [*n* = 30; 15 wood and 15 natural gas (NG) users] who were not part of the cohort but had at least one child < 5 years of age. Informed, signed consent was obtained before every interview. Interviews were conducted in Urdu with all willing participants who could be located and who were willing to participate.

### Exposure data

We ascertained each participant’s type of fuel use, both at the time of interview and during the index pregnancy, and any changes in type of fuel since the pregnancy. The type of stove, cooking area, presence of a chimney**,** and exhaust or window in the kitchen also were ascertained by interview. Reported fuel use during the index pregnancy was also determined by asking the duration of (current) fuel use in years. Cooking frequency, its duration, and type of fuel, and fuel-burning duration per day, number of persons who cooked and for whom cooking was done, and duration of the participant’s stay in the kitchen while fuel was burning were solicited. These interviews were conducted after the survey to provide exposure characterization of fuel use. This included the type of fuel used in index pregnancy and whether participants cooked food during pregnancy. Changes in type of fuel used could be frequent in this setting, as well as changes in duration of cooking, duration of fuel burning, and duration of proximity to burning fuel. Given the strong correlation between socioeconomic status and type of fuel (wood), detailed characterization of socioeconomic status was also necessary. Further lifestyle factors were not considered during the 2000–2002 survey.

### Covariates

We ascertained maternal age in years, marital status, number of family members, house type (straw/mix of straw and bricks/bricks only) and number of rooms, source of water, type of sanitation, monthly income, and occupation and education of spouse and participant. Maternal daytime work and rest duration and the smoking habits of all household members for number, frequency, and duration of use of cigarettes, *beedi* (locally made cigar with tobacco rolled in tobacco leaf), and *huqqa* (burned smoke from charcoal and tobacco, passed through a water pipe and inhaled), inside and outside the house were also obtained. Interview data were also collected regarding frequency, location, and provider of prenatal care, immunizations and illnesses during the index pregnancy, and reproductive and neonatal outcomes.

Trained workers used calibrated scales to measure the participants’ weight and height in light clothing and bare feet at the time of index pregnancy (2000–2002) and at the follow-up interview in 2005. Seca 872 digital scales (Seca, Hamburg, Germany) were used to measure weight with a precision of 100 g. Standard measuring tape was used to measure height with a precision of 10 mm. A Lange skin-fold caliper (Beta Technology Inc., Cambridge, MD, USA) was used to measure left arm triceps skin-fold thickness.

### Data obtained from records

Maternal age, weight at the enrollment during pregnancy, height, gravida status, parity, and any major illness at the time of enrollment of index pregnancy were linked by identification numbers. Maternal interviews (2000–2002) were done by trained workers, and completed forms were checked by field supervisors for errors. Neonatal data included year of birth, infant sex, birth weight in kilograms, crown–heel length in centimeters, head circumference in centimeters, and day of assessment after birth. Gestational age (Parkin et al. 1976), was available for few infants (*n* = 221). Field teams from AKU comprise doctors, nurses, female health visitors, community coordinators, social workers, and locally recruited field workers. These records were obtained by field teams and trained field workers under the supervision of medical officers (doctors). Data were later entered into computer as double data entry under the supervision of a data management unit at AKU.

### Outcome measurements

Field workers informed the field team or medical officers in the field-based health center about all births. Trained field workers measured infant birth weight by a Siltec electronic scale in kilograms (100 g precision), birth length (crown–heel) by a neonatometer (Johnson and Johnson Holtain Ltd., Crymlych, Wales), and head circumference in centimeters by a measuring tape. A random repeat check was done by medical officers on 5% of measurements. Most (91%) neonates were assessed within 48 hr of birth. LBW was defined as birth weight < 2,500 g, and ≥ 2,500 g denoted normal birth weight (NBW). High and low ponderal indices (PIs) were based on greater and less than one standard deviation of mean PI, respectively. PI = 100 × [birth weight in grams/length in cubic centimeters].

### Data management and quality control

We assessed data from records for missing values, errors, and extreme values; extreme values for the upper 95th percentile and lower 5th percentile were verified from original record data. Extreme values of ≥ 60 cm were excluded for the crown–heel length of the neonate (*n* = 6). Interview forms from the surveys done in 2000–2002 and 2004–2005 were checked daily for errors, and data entry was validated by double data entry. All interview forms were coded by the field supervisors before double data entry. After data entry, data were checked and errors corrected by a team of data management unit member, field supervisors, and principal investigator. All field workers were trained and certified in their data collection techniques before they were permitted to collect data in field.

### Data analyses

Continuous variables were summarized with means and standard deviations and checked for normality of distributions. We compared the groups using Student’s *t*-test for continuous variables, and chi-square tests of significance for categorical covariates. Fuel type was the primary independent variable (wood compared with NG); maternal age, body mass index (BMI), gravidity, and socioeconomic status were potential confounding variables. Propensity scores were developed to create probabilities of exposure to certain types of fuel (wood and NG). The propensity scores were calculated primarily to balance the groups and thus adjust simultaneously for many confounding factors while minimizing loss of effective sample size, which would have increased confidence intervals (CIs) if traditional logistic regression models had been used. Interpretation of models is facilitated with one-dimensional propensity scores, which accommodate many more variables than the traditional methods of adjustment. Participants with lower propensity scores were least likely to use wood, as determined by modeling for determinants of wood use. For instance, if a wood user and an NG user have the same propensity score, these two participants would have the same probability to use wood (if they could have the freedom to choose), and hence can be compared on the outcome measurements.

We calculated propensity scores by fitting a logistic regression model to predict the propensity of using wood fuel during the index pregnancy; characteristics associated at *p* < 0.10 were retained in the model. We computed the predicted probability of wood use for each study participant based on the propensity scores. Propensity scores were included in multivariate models to control for differences in covariate distributions between the groups (Rubin 1997).

We estimated a final multiple logistic regression model to determine the association of LBW with wood burning. We tested potentially important variables for demographic, nutritional, reproductive history, lifestyle or habits, and significant socioeconomic status indicators that were related to outcomes for each interaction term. We asssessed interactions by deleting each variable associated with wood use one at a time and then calculating the propensity scores and testing for interactions with wood use and the calculated propensity scores. Odds ratios (ORs) were converted to relative risks (RRs) (Zhang and Yu 1998), and Levin’s population attributable risk (Szklo and Neito 2000) was calculated.

In multiple linear regression models, birth weight and head circumference were regressed on fuel type, propensity scores, and covariates associated with the outcome. Multiple logistic regression modeling evaluated the relation of wood fuel use to high and low PI, with each compared with PI within one SD of the mean.

Data were entered in Epinfo, version 6 (Centers for Disease Control and Prevention, Atlanta, GA, USA), and analyses were conducted using statistical software SPSS version 12.00 for Windows (SPSS Inc., Chicago, IL, USA).

## Results

Compared with those who left the area or refused to participate (*n* = 506), cohort members living in the area (*n* = 806) had a more recent year of birth (*p* < 0.001), infants with smaller head circumference (*p* < 0.04), and early neonatal assessment (*p* < 0.04). The final sample totaled 634 participants, excluding those who reported changes or inconsistent use of single fuel type in the preceding 5 years (*n* = 94), did not cook food during the index pregnancy (*n* = 74), or used fuels other than wood or NG (*n* = 4) (Figure 1).

Record data were missing for crown–heel length (*n* = 12), head circumference (*n* = 6), neonatal assessment day (*n* = 12), maternal gravidity status (*n* = 58), parity (*n* = 154), and BMI (*n* = 7). Compared with infants born to NG users, infants born to wood users had a lower mean birth weight (*p* < 0.06) and higher LBW rate (Table 1). Infants of wood users at birth were also assessed later than those of NG users (*p* < 0.05) (Table 1). Compared with NG users, wood users were older and had higher parity and lower BMI (Table 1).

### Unadjusted results

#### Maternal socioeconomic and cooking characteristics

**Figure 2:**
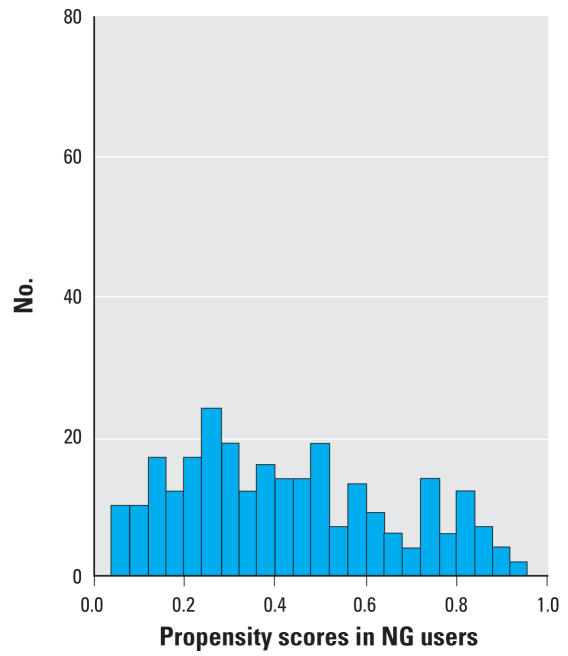
Propensity scores in NG users.

**Figure 3:**
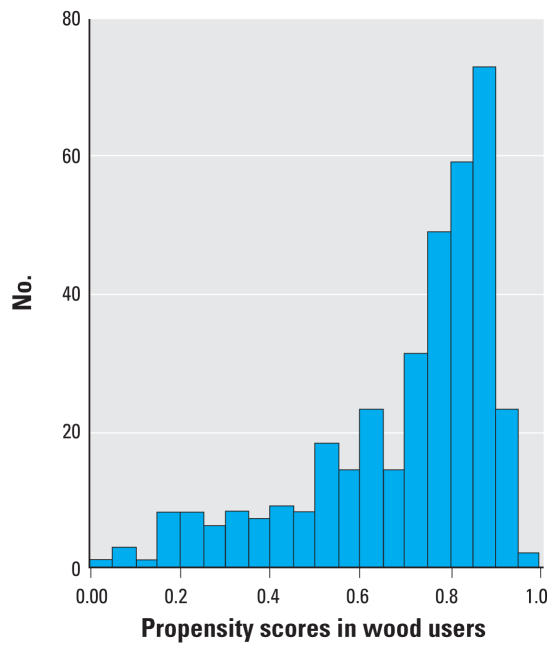
Propensity scores in wood users.

Compared with NG users, significantly more wood users had monthly household incomes less than the median (US$50/month) and lower-quality houses made of a mixture of straw and bricks (Table 1). Households of wood users also had more persons per room, lower maternal and paternal literacy levels, greater frequency of cooking, and more windowless kitchens. Factors associated with wood use in a multiple logistic regression model were absence of a window in the kitchen, having a separate kitchen, spousal and maternal illiteracy, house made of a mixture of straw and bricks, monthly income less than median (Rs. 3,000 ~ US$50), lesser duration of daytime rest during pregnancy, greater gravidity, and lower BMI. The variables used in calculation of propensity scores were taken from the survey done in 2004–2005 because a potentially important variable for parity was missing for many participants from the data obtained in the 2000–2002 survey. The distributions of propensity scores in wood and NG users differed but provided sufficient range of propensity scores in both groups to control for them in multivariate modeling (Figures 2 and 3).

#### Unadjusted results for LBW

Overall, wood use was associated with LBW (RR = 1.51; 95% CI, 1.07–2.12). Prenatal care by doctor had a higher relative risk of LBW compared with that provided by a traditional birth attendant (RR = 1.50; 95% CI, 1.08–2.09); this could be attributed to specific medical needs requiring medical attention during pregnancy. Low maternal BMI at the time of enrollment (2000–2002) and at the time of interview (2004–2005) were significantly associated with delivering an LBW infant.

#### Cooking characteristics related to LBW

A large number of participants reported cooking frequency as more than twice per day and duration of cooking as 180 min (medians); and stratifying data on medians did not result in exactly 50% of data by fuel type. Less frequent cooking was associated with higher RR for LBW among wood (RR = 1.44; 95% CI, 0.78–2.67) and NG users (RR = 1.42; 95% CI, 0.79–2.67), and longer duration of cooking was significantly protective for LBW among NG users (RR = 0.52; 95% CI, 0.30–0.91) but not among wood users (RR = 0.87; 95% CI, 0.58–1.29). Compared with NG users, wood users had a significantly increased RR of LBW for greater frequency of cooking (RR = 1.60; 95% CI, 1.06–2.43), longer duration of fuel burning (RR = 1.57; 95% CI, 1.06–2.33), maternal duration of stay in the kitchen during fuel burning (RR = 1.66; 95% CI 1.03–2.68), and longer duration of cooking (RR = 1.87; 95% CI, 1.17–2.97).

#### Anthropometric outcomes of infants

Unadjusted analyses showed that low (less than the median, 21.7 kg/m^2^) maternal BMI (*p* < 0.001), maternal age < 25 years (*p* < 0.05), history of prenatal tobacco use (*p* < 0.03), maternal smoking (*p* < 0.08), wood use (*p* < 0.06), lower frequency of cooking per day (*p* < 0.08), higher crowding index (*p* < 0.06), infant assessment within 48 hr of birth and birth year before 2002 were related to lower mean birth weight. Mean birth weights tended to increase from 2000 to 2002 (*p* < 0.07). Lower crown–heel length of the infant was associated with younger maternal age (*p* < 0.005), less time spent cooking (*p* < 0.03), lower gravidity (*p* < 0.01), lower maternal BMI (*p* < 0.06), female infant (*p* < 0.05), infant assessment within 48 hr of birth (*p* < 0.001), and infants born after 2000 (*p* < 0.002). Lower head circumference was associated with paternal illiteracy (*p* < 0.07), assessment after 48 hr after birth (*p* < 0.002), being born after 2000 (*p* < 0.002), and higher frequency of cooking per day (*p* < 0.06). Lower PI was significantly associated with maternal tobacco use in pregnancy (*p* < 0.006) and births before 2002 (*p* < 0.002), and insignificantly with maternal illiteracy (*p* < 0.11). Female infants had lower mean birth weight and length, compared with male infants, but head circumference and PI did not differ by sex.

When maternal weight was ≤ 53.8 kg, the mean birth weight of the infant was lower in wood users, although when maternal weight was > 53.8 kg, mean birth weight of neonates did not differ significantly by fuel type. When maternal height was < 151 cm, wood users had infants with lower mean birth weights, whereas birth weight was equivalent in wood and NG users when maternal height was ≥ 151 cm.

NG users had 11.2% (23 of 204) and wood users had 15.5% (42 of 270) of their infants with a lower PI (*p* < 0.11). Among wood users whose infants were LBW, 52.2% (22 of 42) had lower PI (*p* < 0.08), compared with 30% (7 of 23) in NG users.

### Adjusted results

In the final multiple logistic regression model to estimate the change in odds of LBW in infants born to wood users compared with NG users, the beta coefficient for the OR was 0.50, and on inclusion of propensity scores it changed to 0.57. Further adjustment for prenatal examination in hospital, assessment day of newborn, maternal BMI, and parity and gravidity improved the model fit (Table 2). The adjusted OR for LBW associated with wood use was 1.86 (95% CI, 1.11–3.14), which was converted to an RR of 1.64 (95% CI, 1.10–2.34). The increase in the OR after adjustment was caused by negative confounding by gravidity; increasing gravidity is protective of LBW in our data, consistent with existing literature (e.g., Hirve and Ganatra 1994; Lekea-Karanika et al. 1999; Mavalankar et al. 1992) Because wood users tended to have higher gravidity, due to the negative association of parity or gravidity with LBW, adjustment for gravidity in the propensity score resulted in an increase in the OR. The population-attributable risk for LBW associated with wood use, using 50% prevalence of wood use in the population, was 24%; and with a prevalence of 70% of wood and biomass use, it was 31%.

Mean birth weight was significantly lower in wood users (96 g less compared with NG users, *p* < 0.008) in a linear regression model, unadjusted for propensity scores. However, a nonsignificant difference was observed when the propensity score was added to the model (mean reduction of 82 g, *p* < 0.078) (Table 3).

The regression models showed that a later assessment of birth weight was associated with higher birth weight, and prenatal care was related to LBW. Table 4 shows the relationship between fuel type and birth weight stratifying on reported prenatal care during the index pregnancy, on the assumption that those who reported prenatal care at a hospital or clinic had complicated deliveries and were weighed on the same day of birth (delivering at hospital), whereas those who did not report having prenatal care at hospital or clinic could have been weighed later after birth.

## Discussion

In this study we found that exposure to wood fuel burning for cooking during pregnancy was associated with a significantly increased odds of LBW (OR = 1.86) and a reduced mean birth weight of infants (by 82 g), adjusting for potential covariates. More frequent cooking was associated with a higher mean birth weight, possibly because the families that were better off economically could afford to cook more times per day. But increased time spent in the kitchen during fuel burning for cooking was associated with a higher risk of LBW in wood users, though not in NG users. The contribution of wood smoke pollutants may not appear to be substantial in the presence of established nutritional, reproductive, lifestyle, and socioeconomic risk factors, but fuel used for cooking is potentially much more modifiable than these other factors. The use of wood fuel for cooking is estimated to be approximately 53% in Pakistan, and the overall use of biomass fuels is > 70%. The population-attributable risk of 24% for LBW indicates that removal of wood fuel exposure from the population has the potential to reduce the risk of LBW from the current 19% rate to 14.4%, which is just below the targeted rate recommended by the WHO (1995).

Our model for mean birth weight included many confounding factors; thus, it is likely that the association of mean birth weight with wood use would have been statistically significant if we had had a larger sample. The results of this study are consistent for the negative effect of use of wood fuel on birth weight with the two other studies that have evaluated the role of wood fuel in reducing mean birth weight (Boy et al. 2000; Mishra et al. 2004). A reduction of 63 g in birth weight in children whose families use wood/coal compared with gas or electricity-based cooking fuel has been reported from Guatemala, where wood use was either as open fire or through a stove attached to chimney (Boy et al. 2000). A reduction of 120 g in birth weight was reported (using documented birth weight only) from Zimbabwe (in a nationally representative sample from a demographic health survey of 1999), where only the main type of fuel was ascertained, although multiple fuel use was also reported to be common practice (Mishra et al. 2004). The overall rate of LBW in the Guatemala study was 18.8% (greater than that reported in national data from Guatemala of 14%) (Boy et al. 2000) and in Zimbabwe was 8.6% (Mishra et al. 2004). Guatemala is at high altitude (2,500 m); most births were hospital-based, and women in remote rural areas were not included in the study. With a sample of 1,717 participants in the Guatemala study and 3,559 participants in the Zimbabwe study, a significant reduction in mean birth weight was associated with verbal recall of wood fuel use. In our study, all participants lived at a sea level, (a coastal semirural area), which is 50 km from Karachi, without direct access to the city by public transport, with no public hospital in the area, and with > 95% of births occurring in homes and assisted by traditional birth attendants. In our study all wood users had open fires with mud or brick stoves, without an attached chimney, and many used other types of biomass fuels along with wood. Changes of fuel type and cooking during pregnancy were not taken into account in the studies from Guatemala and Zimbabwe, but we observed that such changes occurred in some households. We therefore restricted our analyses to those women who consistently used the same fuel type during the index pregnancy and at the time of interview for the present study.

Our model for low PI (indicator of infant wasting) showed insignificant associations with use of wood fuel, although more wood users had infants who had a low PI (disproportionate in weight for the given height) than did NG users. It is likely that wood smoke could affect body proportions, as has been reported for cigarette smoking (Lindley et al. 2000), but these effects have largely been studied in better nourished populations. Maternal undernutrition leads to symmetric growth retardation, whereas undernutrition later in pregnancy could lead to asymmetric fetal proportions (Akram and Arif 2005). A study from Pakistan in 227 hospital-based LBW infants showed that 40% of LBW infants were asymmetric (Akram and Arif 2005). A mean reduction of head circumference of 0.37 cm was shown for lower levels of continued smoking compared with cessation of smoking during pregnancy in a study of Swedish births (Lindley et al. 2000), and in our study the presence of a smoker in the house was associated with reduced mean head circumference by 0.36 cm (*p* < 0.030). Another factor that could reduce head circumference is day of assessment, because initial assessment may be affected by edema or hematoma after birth, though day of assessment did not differ significantly by self-reported maternal smoking during pregnancy. The finding that lower head circumference was associated with increased frequency of cooking and year of birth was somewhat inconsistent with our finding that birth weight increased with increased frequency of cooking (*p* < 0.08) and with later (more recent) year of birth (*p* < 0.07).

The study had several limitations. First, we did not have complete data for gestational age on most pregnancies to enable us to control for it and to evaluate the proportions of preterm births and infants with intrauterine growth retardation in the two fuel groups. Second, we were missing data on some covariates, such as use of prenatal multivitamins or iron supplements and any prenatal illnesses, which could have led to some uncontrolled confounding. The women in our study were followed until the end of pregnancy and were referred by health workers for routine prenatal supplements, including iron and folic acid; in many cases, health workers provided supplements at home, regardless of fuel type. We ascertained and analyzed self-reported anemia, diabetes, hypertension, and other illnesses but, possibly because of inaccuracy of recall, we did not find any important relationships between them and LBW or mean birth weight. Third, because the day of assessment of newborns was later for wood users than for NG users, it is possible that some of the LBW in the wood fuel group was attributed to infants losing weight after birth, as is normal, and having their weight measured later, although the association persisted after adjustments for day of neonatal assessment. The day of measurement of birth weight had an influence (Pearson correlation coefficient = 0.11, *p* < 0.001) on birth weight; this relationship was linear over a longer period of time, and the correlation was statistically significant both parametrically and nonparametrically. However, in the linear regression model increasing birth weight occurred with increasing day of assessment (which occurred more in wood users). Our study sample also included only participants who had live singleton births; births that ended in miscarriages or fetal or early neonatal deaths were excluded. Considering these observations and the high proportion of male infants who had LBW than female infants among NG users, if any bias occurred it was toward the null, thus making our estimates of effects of use of wood fuel conservative.

The present study also had major strengths. First, we used a retrospective, population-based cohort design with a large sample size, so that the temporal sequence of use of wood fuel preceding the occurrence of LBW in a non-clinic-based sample of pregnant women was ascertained. Second, the estimated relative risk was adjusted for multiple demographic, nutritional, reproductive, lifestyle (health-related), and socioeconomic factors, thus minimizing the likelihood of uncontrolled confounding affecting the results. Use of wood fuel and LBW are linked with poverty (Boy et al. 2000; Gupta and Sreevidya 2004; Mishra et al. 2004), and poverty is related to unhealthy and potentially unhealthy behaviors (Gupta and Sreevidya 2004; Kramer et al. 2000). Our analytic techniques controlled for these effects so that the independent effect of use of wood fuel on LBW could be observed. Indeed, the effect of use of wood fuel on LBW increased after accounting for confounding factors and the day of newborn assessment from an OR of 1.65 (95% CI, 1.09–2.88) to an OR of 1.86 (95% CI, 1.13–3.06). As we collected data on many potentially confounding factors, adjusting for all of them individually in the usual logistic regression model might not have accounted sufficiently for all confounding and would have reduced the effective sample size, thus increasing the confidence intervals. The strong correlation between socioeconomic status in particular and use of wood was thus better addressed by using propensity scores. We also conducted traditional logistic regression models that included the individual variables and found the results to be quite similar, with point estimates being very close and in the same direction but with CIs being wider for most variables.

This large population-based study adds to the growing body of evidence concerning the effects of wood use on risk of LBW and corroborates the known associations of maternal nutrition, reproductive, and socioeconomic factors with infant birth weight. Notably, accounting for differences in demographic, socioeconomic, and nutrition factors further strengthened the validity of our findings that associated prenatal wood fuel use with increased risk of LBW. Thus, preventive measures designed to minimize or remove this exposure could dramatically reduce the occurrence of LBW and thus the morbidity and mortality that results from LBW.

## Figures and Tables

**Table 1:** Maternal and neonatal characteristics by type of fuel.

Characteristic	NG (*n* = 268)	Wood (n = 366)	*p*-Value
Maternal demographic and nutritional status 2000–2002 (mean ± SD)			
Maternal age (years)	24.70 ± 4.65	25.38 ± 4.94	0.08
Maternal parity	2.19 ± 1.9	2.71 ± 2.1	0.002
Maternal gravidity	3.28 ± 1.9	3.80 ± 2.2	0.003
Maternal BMI (kg/m^2^)	22.30 ± 3.37	21.81 ± 3.0	0.05
Data from 2005 (mean ± SD)			
Maternal age (years)	29.10 ± 5.0	30.26 ± 5.8	0.009
Maternal parity	4.3 ± 2.2	4.64 ± 2.5	0.09
Maternal gravidity	4.69 ± 2.5	5.09 ± 2.7	0.06
Maternal BMI (kg/m^2^)	21.11 ± 3.97	20.11 ± 2.8	0.001
Reported socioeconomic status for index pregnancy			
Crowding index (mean ± SD)	4.40 ± 2.09	4.70 ± 2.19	0.07
Monthly income > median [> $50 (%)]	35.5	20.2	0.001
Type of house construction (%)			
Straw only	0.03	23.5	0.001
Mix of straw and bricks/tin and bricks	71.6	65.8	
Bricks only	25.4	10.7	
Mother literate (%)	20.5	11.7	0.002
Spouse literate (%)	32.1	20.5	0.001
Spouse work (%)			0.08
Unskilled labor	19.0	13.7	
Fisherman	75.0	82.2	
Other skilled/office worker	06.0	04.0	
Reported antenatal care in index pregnancy			
Prenatal checks (%)			0.27
None	37.7	34.2	
Home	10.8	15.0	
Clinic/hospital/health center	51.5	50.8	
Day time rest [min (mean ± SD)]	75.0 ± 69.2	62.2 ± 58.3	0.01
Reported maternal and family history of smoking and oral tobacco use (%)			
Maternal smoking	16.8	17.5	0.45
Reported prenatal smoking	14.6	16.1	0.33
Maternal tobacco chewing	28.7	26.8	0.59
Reported prenatal chewing tobacco use	25.7	22.1	0.16
Reported maternal exposure for index pregnancy and birth outcomes from record data			
Cooking frequency/day (mean ± SD)	2.6 ± 0.7	2.7 ± 0.6	0.01
Minutes fuel burned/day (mean ± SD)	190.4 ± 62.4	186.6 ± 47.7	0.38
Window in kitchen (%)	63.1	21.0	0.001
Birth weight [kg (mean ± SD)]	2.84 ± 0.44	2.78 ± 0.45	0.06
Crown–heel length [cm (mean ± SD)]	47.54 ± 2.94	47.28 ± 3.47	0.33
Neonatal assessment day (mean ± SD)	1.37 ± 1.15	1.65 ± 2.03	0.05
Year of birth (% births)			0.20
2000	24.5	29.3	
2001	50.2	43.3	
2002	25.3	27.4	
LBW in neonates (%)	15.0	22.7	0.01
Percent males in neonates	57.1	54.9	0.32

**Table 2:** Multiple logistic regression models for LBW (< 2,500 g).

Variable	*p*-Value	OR (95% CI)
Model 1		
Type of fuel in pregnancy: wood	0.01	1.65 (1.09–2.51)
Constant	0.00	0.17
Model 2		
Type of fuel in pregnancy: wood	0.02	1.77 (1.09–2.88)
Increasing propensity scores^a^	0.61	0.79 (0.31–1.97)
Constant	0.00	0.19
Model 3		
Type of fuel in pregnancy: wood	0.01	1.86 (1.11–3.14)
Prenatal examination at hospital/clinic	0.01	1.71 (1.11–2.63)
Increasing neonatal assessment day	0.05	0.79 (0.62–1.004)
Increasing gravidity (prepregnancy)	0.05	0.89 (0.79–1.003)
Increasing BMI (kg/m^2^) (prepregnancy)	0.00	0.90 (0.83–0.96)
Increasing propensity scores^a^	0.59	0.76 (0.28–2.04)
Constant	0.22	2.87

a Includes variables for having a separate kitchen for cooking, absence of window in kitchen, spouse not literate, mother not literate, straw/straw and brick mix type house construction, less than median monthly income level, increasing duration of daytime sleep or rest during pregnancy, increasing gravidity, and increasing body mass index.

**Table 3:** Multiple linear regression model for mean birth weight (kg).

Variable in model	β	*p*-Value	*t*	95% CI
Use of wood fuel	−0.082	0.07	−1.76	−0.17 to 0.009
Maternal tobacco use in pregnancy	−0.077	0.09	−1.65	−0.16 to 0.014
Maternal smoking in pregnancy	−0.098	0.06	−1.86	−0.20 to 0.005
Increasing maternal age	0.010	0.006	2.77	0.003 to 0.018
Male sex (neonate)	0.080	0.03	2.07	0.004 to 0.157
Frequency of cooking per day	0.054	0.08	1.72	−0.007 to 0.115
Increasing BMI (kg/m^2^) (2000–2002)	0.019	0.002	3.15	0.007 to 0.031
Increasing gravidity (2000–2002)	0.011	0.27	1.08	−0.009 to 0.032
Increasing birth year (2000–2002)	0.072	0.007	2.72	0.02 to 0.124
Increasing day of neonatal assessment after birth	0.033	0.003	3.00	0.01 to 0.054
Increasing propensity scores^a^	−0.057	0.53	−0.62	−0.23 to 0.120
Constant	−142.89	0.007	−2.69	−247.2 to −38.5

a Includes variables for having a separate kitchen for cooking, absence of window in kitchen, spouse not literate, mother not literate, straw/straw and brick mix type house construction, less than median monthly income level, increasing duration of daytime sleep or rest during pregnancy, increasing gravidity, and increasing body mass index.

**Table 4:** Relationship between type of fuel and birth weight stratified on reported prenatal care^a^ in infants weighed on the day of birth.

	No prenatal care reported^b^			Prenatal care reported^c^		
Fuel type	LBW (no.)	NBW (no.)	Birth weight (kg) (mean ± SD)	LBW (no.)	NBW (no.)	Birth weight (kg) (mean ± SD)
Wood	33	109	2.74 ± 0.44	36	106	2.77 ± 0.47
NG	11	98	2.87 ± 0.40	23	86	2.76 ± 0.44

a Reported prenatal care, as a proxy for complicated deliveries measured soon after birth.

b RR = 2.30 (95% CI, 1.22–4.35); *p* = 0.01.

c RR = 1.20 (95% CI, 0.76–1.90); *p* = 0.42.
